# A Two-Phase Embedding Approach for Secure Distributed Steganography

**DOI:** 10.3390/s25051448

**Published:** 2025-02-27

**Authors:** Kamil Woźniak, Marek R. Ogiela, Lidia Ogiela

**Affiliations:** 1 Cryptography and Cognitive Informatics Laboratory, AGH University of Krakow, 30 Mickiewicza Avenue, 30-059 Kraków, Poland; kamilwozniak@agh.edu.pl; 2 Faculty of Computer Science, AGH University of Krakow, 30 Mickiewicza Avenue, 30-059 Kraków, Poland; logiela@agh.edu.pl

**Keywords:** cybersecurity, distributed steganography, secret hiding, secret sharing

## Abstract

Steganography serves a crucial role in secure communications by concealing information within non-suspicious media, yet traditional methods often lack resilience and efficiency. Distributed steganography, which involves fragmenting messages across multiple containers using secret sharing schemes, offers improved security but increases complexity. This paper introduces a novel two-phase embedding algorithm that mitigates these issues, enhancing both security and practicality. Initially, the secret message is divided into shares using Shamir’s Secret Sharing and embedded into distinct media containers via pseudo-random LSB paths determined by a unique internal stego key. Subsequently, this internal key is further divided and embedded using a shared stego key known only to the sender and receiver, adding an additional security layer. The algorithm effectively reduces key management complexity while enhancing resilience against sophisticated steganalytic attacks. Evaluation metrics, including Peak Signal-to-Noise Ratio (PSNR) and Structural Similarity Index Measure (SSIM), demonstrate that stego images maintain high quality even when embedding up to 0.95 bits per pixel (bpp). Additionally, robustness tests with StegoExpose and Aletheia confirm the algorithm’s stealthiness, as no detections are made by these advanced steganalysis tools. This research offers a secure and efficient advancement in distributed steganography, facilitating resilient information concealment in sophisticated communication environments.

## 1. Introduction

Steganography, the art and science of hiding information, plays a pivotal role in secure communications by concealing the existence of a message within various non-suspicious media such as images, audio, video, or text files. The primary objective of steganography is to hide the existence of the communication, which differentiates it from cryptography, which only obscures the content of the message [1,2]. Widely used techniques include the Least Significant Bit (LSB) method, frequency domain embedding using the Discrete Cosine Transform (DCT), and methods using the Discrete Wavelet Transform (DWT) [3,4,5]. More recently, methods that employ machine learning and deep neural networks have gained prominence [5,6,7,8]. Applications range from managing digital rights and confidential data storage to protecting military and government communications [9,10]. With digital communication growing exponentially, there is an increasing need for robust steganographic methods to safeguard privacy and enhance data security over open networks.

Despite recent advances, contemporary steganography faces three critical limitations: (1) embedding in a single container creates concentrated statistical anomalies that modern steganalysis tools often detect; (2) distributed methods generally require complex key management; and (3) security enhancements typically compromise imperceptibility. Traditionally, embedding a secret message within one container exposes it to detection and limits the concealment capacity [11]. Although various innovative techniques have emerged, many still lack robustness and broad applicability, reducing their effectiveness in advanced scenarios. Furthermore, the development of detection tools that employ techniques such as statistical analysis, linear analysis, and wavelet statistics further challenges the resilience of these methods [3]. Consequently, there is a pressing need for more secure and adaptable steganographic approaches that can withstand these detection techniques and provide enhanced protection.

Researchers have explored distributed steganography, which involves hiding message fragments in multiple media containers. This approach serves to improve security by mitigating the risk of detection and, moreover, increases the resilience and redundancy of concealed information [12,13,14,15,16,17]. To achieve this, secret sharing schemes, such as the most popular Shamir secret sharing protocol, can be used to divide a secret into multiple shares, thus ensuring that only a sufficient number of shares can reconstruct the original message [18,19,20]. This integration helps to improve the security and redundancy of steganographic methods by distributing the secret between multiple containers [21,22].

However, existing distributed methods exhibit critical shortcomings. They require separate keys for each media container, remain vulnerable to the localized steganalysis of individual shares, and suffer from capacity fragmentation that reduces the usable payload. A fundamental trade-off arises when using secret sharing in distributed steganography: increasing the number of shares (to enhance security through redundancy) requires embedding data in more containers, which proportionally increases the total payload and increases detection risk. For example, distributing a secret into *n* shares requires embedding *n* fragments, multiplying the cumulative payload by a factor of *n* compared to single-container embedding. Conversely, reducing the number of shares simplifies embedding and lowers detection likelihood but weakens security and redundancy, as fewer shares are required for reconstruction. This balance is critical: higher security amplifies exposure through greater payload distribution, while lower security risks compromising the integrity of the concealed message. As highlighted in [23], optimizing steganography requires balancing four competing aspects: imperceptibility (minimizing detectability), security (resisting extraction), payload (maximizing capacity), and robustness (withstanding distortions). Existing methods often prioritize one aspect at the expense of others; for instance, robustness-enhancing redundancy reduces payload, while high imperceptibility can weaken security. Distributed steganography partially addresses this but introduces fragmented payloads and key management complexity.

To resolve these trade-offs, we propose a two-phase embedding mechanism that integrates secret sharing with dual-key isolation. In the first phase, the secret message is divided into multiple shares using a threshold-based secret sharing scheme; each share is embedded into a distinct media container using a randomly generated internal stego key. In the second phase, this internal stego key is partitioned into fragments (through secret sharing) and embedded into the corresponding containers using a shared stego key known only to the sender and receiver. This approach uniquely addresses the four critical aspects: (1) security is improved by distributing secrets across containers, reducing localized statistical anomalies; (2) payload is preserved by isolating key fragments from message shares, avoiding capacity fragmentation; (3) imperceptibility is maintained through lightweight spatial-domain embedding (PSNR > 51 dB, SSIM > 0.99); and (4) robustness is improved through threshold-based reconstruction, tolerating partial share loss.

The novelty of our approach lies in the integration of secret sharing with a two-phase embedding strategy. Experimental validation on 3000 BOSSBase images demonstrates its effectiveness. Using the StegExpose [24] and Aletheia framework [25], our method achieves a 0% detection rate at a capacity of 0.95 bits per pixel (bpp), compared to a 32% detection rate for the traditional LSB approach.

This paper is organized as follows. Section 2 reviews the background and related work, while Section 3 details our proposed two-phase embedding algorithm. Section 4 presents a theoretical analysis of the algorithm’s security, embedding capacity, and efficiency. The evaluation and results of the algorithm against known steganographic attacks and imperceptibility analysis are presented in Section 5. In Section 6, we interpret our findings, explore their implications for the field, and discuss potential applications and limitations. Finally, we conclude by summarizing our key contributions and suggesting directions for future research in Section 7.

## 2. Background and Related Work

Steganography, derived from the Greek words *steganos* (meaning “covered”) and *graphia* (meaning “writing”), is the practice of hiding a secret message within an ordinary, non-secret, non-suspicious medium, called a cover medium, to avoid detection by unauthorized recipients [1,2,26]. Unlike cryptography, which only hides the content of a message, steganography hides the very existence of the message, thus providing an additional layer of security [2]. Cryptography can and often is combined with steganography, but it is important to distinguish between them. The exponential growth of digital communication and the Internet has increased the importance of steganography, especially in applications that require covert information exchange, such as secure military communications, confidential corporate correspondence, and digital rights management [9].

Steganographic techniques have evolved considerably, and they can be broadly categorized based on the media used for embedding (e.g., images, audio, video, and text) and the methods employed for the embedding process. Among the most widely used methods in image steganography is the Least Significant Bit (LSB) substitution, where the LSB of each pixel’s color value in an image is replaced with bits from the secret message [3,4]. Although simple and with high embedding capacity, LSB methods are vulnerable to statistical and visual attacks due to predictable embedding patterns. Some implementations of the LSB scheme also use pseudo-random paths (based on shared stego key) and adaptive techniques to embed message bits [27,28].

To improve robustness and imperceptibility, steganographic methods have been developed in the transform domain. These techniques embed secret messages into the frequency components of the cover media rather than directly altering pixel values. They exploit the lower sensitivity of the human visual system to high-frequency changes, thus concealing information in areas where perceptual differences are minimal [4]. Transform domain techniques, such as those based on the Discrete Cosine Transform (DCT) [4,5,28,29] and the Discrete Wavelet Transform (DWT) [28,30,31,32], offer improved robustness against detection and various image processing attacks by spreading the embedded data across transformed coefficients, making alterations less perceptible.

More recent approaches utilize machine learning and deep learning techniques, including artificial neural networks, to optimize the embedding process, improve security, and resist steganalytic attacks [5,6,7,8,33]. For example, deep neural networks can identify optimal embedding locations or generate stego media that are statistically indistinguishable from the original cover [6,7]. In addition, secret-to-image transformation methods based on Generative Adversarial Networks (GANs) have emerged, whereby a cover image is generated from secret information itself [8,34,35,36].

Despite these advances, traditional methods that embed secret messages within a single media container still face significant challenges. One primary concern is vulnerability to steganalytic methods, which exploit statistical anomalies introduced during embedding, particularly when predictable patterns or significant alterations in the statistics of the cover medium occur [37,38].

Furthermore, single-media steganography poses a substantial security risk due to its reliance on a single point of failure. If the stego container is intercepted, lost, or corrupted, the entire secret message can be compromised or rendered unrecoverable [11]. Moreover, the embedding capacity is inherently limited; overloading a single container with excessive hidden data increases the risk of noticeable artifacts and detection [3].

To address these limitations, researchers have explored distributed steganography, which divides a secret message into multiple fragments and embeds each fragment in separate cover media [12,13,14,15,16,17]. This strategy not only mitigates the risk associated with a single point of failure but also complicates an adversary’s efforts to reconstruct the entire secret without access to multiple stego media [21,22].

Distributed steganography often employs secret sharing schemes, which split a secret into multiple parts (shares) such that only a specific combination can reconstruct the original message. Shamir’s Secret Sharing algorithm is the most notable example of this approach, where any *k* of the *n* shares (with k≤n) can reconstruct the secret, while fewer than *k* shares reveal no information [18]. Over time, more sophisticated threshold schemes have been developed [19,20], including those based on cellular automata [39].

By integrating secret sharing with steganography, each share is embedded in a different cover medium, thereby increasing security and robustness through the following:

- Improved resilience: The loss or interception of some stego media does not compromise the entire secret, as long as the required threshold is met.
- Enhanced security: An adversary must obtain and correctly extract multiple stego media to reconstruct the secret, significantly increasing the complexity and resources required for an attack [12,21].

However, distributed steganography faces challenges. Embedding secret shares in multiple cover media often requires managing multiple stego keys or parameter—one for each medium—increasing system complexity and the burden of secure key distribution [40]. Moreover, advanced steganalysis techniques capable of correlating patterns in multiple media can still detect anomalies if the embedding process introduces consistent artifacts [22].

Given these challenges, it is evident that current distributed steganography techniques are not fully equipped to meet the evolving security requirements of modern communication environments. Simplifying key management is essential to reduce system complexity and improve usability, making it more practical for real-world applications. Moreover, the development of methods that are resistant to advanced steganalysis while maintaining high embedding capacity and imperceptibility constitutes a significant gap in the existing literature.

Motivated by these issues, our research proposes a novel distributed steganography algorithm that reduces key complexity, improves security, and enhances robustness against sophisticated steganalytic attacks. Our innovative two-phase embedding mechanism integrates secret sharing schemes with a dual-layer embedding approach that distributes both the secret message and key information across multiple media containers in a secure and efficient manner. This solution aims to bridge existing gaps in distributed steganography and contribute a practical method for modern secure communication infrastructures.

## 3. Proposed Algorithm

In this section, we introduce our innovative distributed steganography algorithm designed to enhance security, robustness, and practicality in obscuring information by distributing it across multiple media carriers. This general-purpose framework is applicable to a variety of media containers and steganographic embedding techniques, allowing adaptation to different use cases and media types such as images, audio files, video files, and text documents.

The primary innovation of the proposed algorithm is its two-phase embedding mechanism, which integrates secret sharing schemes with a dual-layer embedding process. This approach distributes both the secret message and the key information required for its extraction across multiple carriers, thereby adding an extra layer of protection against steganalytic attacks.

The detailed framework of the embedding procedure is presented in Section 3.1, with the secret retrieval process described in Section 3.2. Section 3.3 discusses potential interference issues between embedding functions, and Section 3.4 provides a proposed implementation using proportional non-overlapping regions.

### 3.1. Embedding Procedure

The embedding process is organized into two distinct phases, each designed to improve the security of the concealed information while offering flexibility for different media and embedding techniques. It is essential to select the embedding functions and their parameters to avoid any overlap between the two phases, which could corrupt the embedded data.

#### 3.1.1. Phase 1: Embedding of the Secret Message

**Figure 1:**
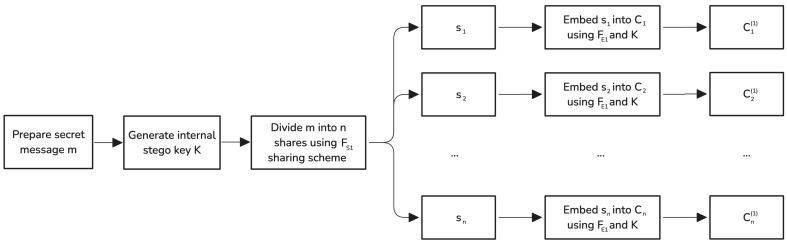
Flow chart of the first phase of the algorithm for embedding a secret message *m* into *n* cover containers $C_{i}$ using embedding function $F_{E1}$ and internal stego key *K* to generate a set of intermediate stego containers.

In the first phase, the confidential message, denoted by *m*, is divided into *n* shares using a predetermined (t,n)-threshold secret sharing scheme [18,19,20]. Each share is then embedded into a separate media container using an arbitrarily selected internal stego key *K*. Figure 1 shows the flow diagram of this phase. The steps are as follows:

1. Secret message preparation: The sender prepares the secret message *m* for embedding. The preprocessing steps may include encryption, compression, or formatting to optimize the message for secure and efficient embedding. Encrypting *m* prior to the secret sharing process adds an additional layer of security, especially if the message contains highly sensitive information.
2. Generation of the internal stego key: A random internal stego key *K* is generated. This key is used consistently across all containers in this phase, simplifying key management and preparing for secure key distribution in the subsequent phase.
3. Dividing the secret message: The secret message *m* is divided into *n* shares $\left\{s_{1},s_{2},\cdots ,s_{n}\right\}$ using a (t,n)-threshold secret sharing scheme $F_{S1}$:
  (1) $$\left\{s_{1},s_{2},\cdots ,s_{n}\right\}=F_{S1}\left(m\right).$$
4. Embedding shares into selected containers: Each message share $s_{i}$ is embedded into the corresponding media container $C_{i}$ using an embedding function $F_{E1}$ and the internal stego key *K*:
  (2) $$C_{i}^{\left(1\right)}=F_{E1}\left(C_{i},s_{i},K\right),$$
  for i=1,2,⋯,n, where $C_{i}^{\left(1\right)}$ denotes the stego container after Phase 1.

The embedding function $F_{E1}$ can be any method suitable for the cover media and security requirements. However, it is imperative that $F_{E1}$ ensures that the embedding locations do not overlap with those used in Phase 2. For example, $F_{E1}$ can employ an LSB-based technique with a pseudo-random embedding path derived from the internal stego key [26]. Regardless of the specific embedding strategy, it is crucial to enforce non-overlapping embedding regions in order to prevent interference and guarantee the reliable retrieval of both the hidden message fragments and the internal stego key.

Upon completion of Phase 1, the sender receives a set of intermediate stego containers $\left\{C_{1}^{\left(1\right)},C_{2}^{\left(1\right)},\ldots ,C_{n}^{\left(1\right)}\right\}$, each containing a share of the secret message embedded using an internal stego key *K*.

#### 3.1.2. Phase 2: Embedding of the Internal Stego Key

**Figure 2:**
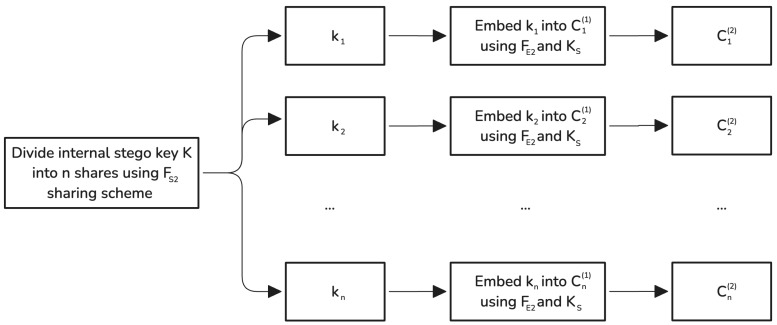
Flowchart of the second phase of the algorithm for distributing the internal stego key *K* across *n* stego containers using embedding function $F_{E2}$ and shared stego key $K_{S}$ to generate a set of the final stego containers.

In Phase 2, the internal stego key *K* generated in Phase 1 is secured by splitting it into *n* key shares and embedding each share into the corresponding stego container $C_{i}^{\left(1\right)}$ using a shared stego key $K_{S}$ that is known only to the sender and receiver. Figure 2 illustrates this phase. The steps are as follows:

1. Dividing the internal stego key: The internal stego key *K* is divided into *n* key shares $\left\{k_{1},k_{2},\cdots ,k_{n}\right\}$ using a $\left(t^{\prime },n\right)$-threshold secret sharing scheme $F_{S2}$, which can be the same as or different from the scheme $F_{S1}$ used in Phase 1. This process can be represented as
  (3) $$\left\{k_{1},k_{2},\cdots ,k_{n}\right\}=F_{S2}\left(K\right).$$
  The threshold $t^{\prime }$ (with $t^{\prime }\geq t$) can be chosen independently of *t* from Phase 1. This allows for the establishment of security parameters that are more flexible and tailored to specific requirements.
2. Embedding key shares into stego containers: Each key share $k_{i}$ is embedded in the corresponding stego container $C_{i}^{\left(1\right)}$ from Phase 1 using an embedding function $F_{E2}$ and the shared stego key $K_{S}$:
  (4) $$C_{i}^{\left(2\right)}=F_{E2}\left(C_{i}^{\left(1\right)},k_{i},K_{S}\right),$$
  for i=1,2,⋯,n, where $C_{i}^{\left(2\right)}$ is the final stego container after Phase 2.

The embedding function $F_{E2}$ can be any steganographic method appropriate for the type of media and security requirements, and may differ from $F_{E1}$ used in Phase 1. Regardless of the method selected for $F_{E2}$, it must embed key shares in locations that do not overlap with regions modified by $F_{E1}$. This requirement can be fulfilled by partitioning the cover media or by ensuring that the pseudo-random embedding paths used by $F_{E1}$ and $F_{E2}$ are disjoint. In this way, the data embedded during Phase 1 remain isolated from the key shares introduced in Phase 2, preserving both security and recoverability.

Upon the completion of Phase 2, the secret message and the internal stego key (necessary for retrieval) are securely distributed across the final stego containers $\left\{C_{1}^{\left(2\right)},C_{2}^{\left(2\right)},\cdots ,C_{n}^{\left(2\right)}\right\}$. An adversary would need to access at least $t^{\prime }$ containers and possess $K_{S}$ to reconstruct *K*, and at least *t* message shares to recover the secret message *m*.

### 3.2. Secret Retrieval Procedure

Given any $t^{\prime }$ of the *n* final stego containers $\left\{C_{i_{1}}^{\left(2\right)},C_{i_{2}}^{\left(2\right)},\cdots ,C_{i_{t^{\prime }}}^{\left(2\right)}\right\}$, where $i_{j}\in \left\{1,2,\ldots ,n\right\}$ and all $i_{j}$ are distinct, and the shared stego key $K_{S}$, the receiver can retrieve the original secret message *m* by reversing the processes of both embedding phases. The receiver can reconstruct the original secret message *m* by reversing both embedding phases:

1. Extraction of key shares: For each received stego container $C_{i_{j}}^{\left(2\right)}$, the receiver extracts the key share $k_{i_{j}}$ using the extraction function $F_{E2}^{-1}$ and the shared stego key $K_{S}$:
  (5) $$k_{i_{j}}=F_{E2}^{-1}\left(C_{i_{j}}^{\left(2\right)},K_{S}\right),$$
  for $j=1,2,\ldots ,t^{\prime }$, the function $F_{E2}^{-1}$ is the inverse of the embedding function $F_{E2}$ used in Phase 2 of the embedding procedure.
2. Reconstruction of the internal stego key: Using the extracted key shares $\left\{k_{i_{1}},k_{i_{2}},\cdots ,k_{i_{t^{\prime }}}\right\}$ and the secret sharing reconstruction function $F_{S2}^{-1}$, the internal stego key *K* is reconstructed:
  (6) $$K=F_{S2}^{-1}\left(\{k_{i_{1}},k_{i_{2}},\cdots ,k_{i_{t^{\prime }}}\}\right).$$
3. Extraction of message shares: With the internal stego key *K*, the receiver uses the inverse function $F_{E1}^{-1}$ on each corresponding container $C_{i_{j}}^{\left(2\right)}$ to extract the message share $s_{i_{j}}$:
  (7) $$s_{i_{j}}=F_{E1}^{-1}\left(C_{i_{j}}^{\left(2\right)},K\right),$$
  for j=1,2,⋯,t. The function $F_{E1}^{-1}$ operates on $C_{i_{j}}^{\left(2\right)}$ and retrieves the embedded message share $s_{i_{j}}$ using the internal stego key *K*. Since $F_{E1}$ and $F_{E2}$ are designed to use non-overlapping embedding locations, the data embedded in Phase 1 remain intact and can be accurately extracted without interference from the data embedded in Phase 2.
4. Reconstruction of the secret message: Finally, using the collected message shares $\left\{s_{i_{1}},s_{i_{2}},\cdots ,s_{i_{t}}\right\}$ and the reconstruction function $F_{S1}^{-1}$, the original secret message *m* is recovered:
  (8) $$m=F_{S1}^{-1}\left(\{s_{i_{1}},s_{i_{2}},\cdots ,s_{i_{t}}\}\right).$$

This layered retrieval procedure ensures that, without at least $t^{\prime }$ containers and the shared stego key $K_{S}$, an adversary cannot reconstruct *K*; furthermore, *m* remains unrecoverable without *t* valid message shares.

### 3.3. Embedding Function Interference Problem

A potential challenge in the proposed algorithm is the interference between the two embedding phases within the same media container. If $F_{E1}$ and $F_{E2}$ embed data in overlapping regions, data from one phase can overwrite or corrupt data from the other, preventing successful retrieval of the secret message or the internal stego key.

For example, if both functions utilize the LSB method with pseudo-random embedding paths determined by *K* and $K_{S}$, overlapping locations can lead to corruption [26]. To avoid this, it is critical that the embedding functions operate on non-overlapping regions. This problem can be resolved by carefully designing the functions, distributing separate embedding regions, or employing different techniques or domains for each phase. Section 3.4 illustrates a solution using proportional, non-overlapping regions with pseudo-random embedding paths.

### 3.4. Proposed Implementation Using Proportional Non-Overlapping Embedding Regions

To demonstrate how non-overlapping regions prevent interference, we provide an implementation using grayscale image steganography with the Least Significant Bit (LSB) method. Both embedding functions $F_{E1}$ and $F_{E2}$ utilize the LSB technique with key-driven pseudo-random embedding paths determined by their respective stego keys *K* and $K_{S}$ [26].

Suppose that each grayscale cover image $C_{i}$ has dimensions M×N, and that all images have the same dimensions. We denote the set of pixel indices in $C_{i}$ by *P*, where |P|=M×N. For computational convenience, *P* is considered a flat list of indices ranging from 1 to |P|. Each index p∈P corresponds to a pixel location in the image $C_{i}$.

To prevent interference, the pixels are divided into two distinct, non-overlapping regions:

- Region A: used exclusively by $F_{E1}$ in Phase 1 to embed message shares $\left\{s_{1},s_{2},\cdots ,s_{n}\right\}$.
- Region B: used exclusively by $F_{E2}$ in Phase 2 to embed key shares $\left\{k_{1},k_{2},\cdots ,k_{n}\right\}$.

**Figure 3:**
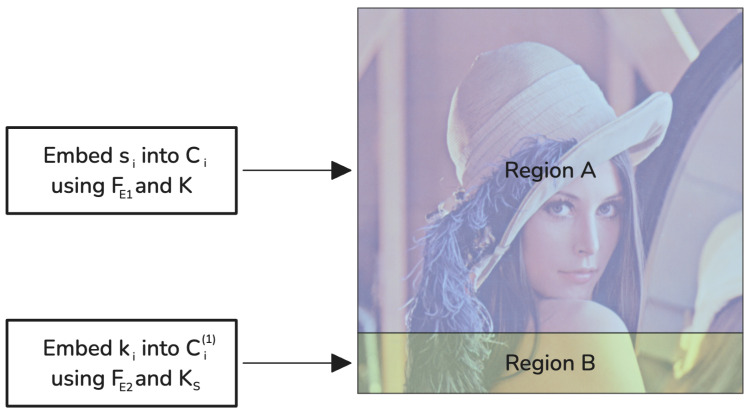
Illustration of proportional non-overlapping embedding regions in the proposed implementation.

Given that the size of *K* is much smaller than that of *m*, Region B occupies a much smaller portion of the cover image compared to Region A. The division of the set of pixel indices *P* into Region A and Region B is based on the proportion of data sizes. Let $S_{m}$ be the size (in bits) of the message share $s_{i}$, and $S_{k}$ be the size (in bits) of the key share $k_{i}$. In our implementation, cryptographic hashing is used to define non-overlapping regions while preserving payload proportionality. For each pixel index *p*, we compute

(9) $$H\left(p,K_{S}\right)=\mathrm{SHA}3\left(p\|K_{S}\right)\;\mathrm{mod}\;\left(S_{m}+S_{k}\right),$$

where SHA3 is the Secure Hash Algorithm 3 [41], and ‖ denotes concatenation. Pixels are assigned to Region A if $H\left(p,K_{S}\right)<S_{m}$, and to Region B otherwise. Figure 3 illustrates the division of the cover image into two proportional, non-overlapping regions.

Subsequently, pixels from each region are permuted using a cryptographically secure PRNG seeded with *K* (for Region A) or $K_{S}$ (for Region B) to generate pseudo-random embedding paths:

(10) $${\mathrm{Path}}_{A}=\mathrm{PRNG}\left(\mathrm{Region}\;A,K\right),$$

(11) $${\mathrm{Path}}_{B}=\mathrm{PRNG}\left(\mathrm{Region}\;B,K_{S}\right),$$

where ${\mathrm{Path}}_{A}$ and ${\mathrm{Path}}_{B}$ are the respective shuffled pixel sequences. The embedding then follows these paths sequentially for each cover container:

- Phase 1: embedding of the secret message share:
  – The embedding function $F_{E1}$ utilizes the shared stego key $K_{S}$ to identify an embedding Region A and employs an internal stego key *K* to generate a pseudo-random path within it.
  – The message share $s_{i}$ is embedded in the Least Significant Bits of the pixels along ${\mathrm{Path}}_{A}$ in the container $C_{i}$, resulting in the stego container $C_{i}^{\left(1\right)}$.
- Phase 2: embedding of the internal stego key share:
  – The embedding function $F_{E2}$ utilizes the shared stego key $K_{S}$ to identify an embedding Region B and to generate a pseudo-random path within it.
  – The key share $k_{i}$ is embedded into the Least Significant Bits of the pixels along ${\mathrm{Path}}_{B}$ in $C_{i}^{\left(1\right)}$, producing the final stego container $C_{i}^{\left(2\right)}$.

By allocating embedding regions proportionally based on $S_{m}$ and $S_{k}$, we maximize the cover image’s capacity while preventing interference between embedding phases. Because $S_{k}\ll S_{m}$, Region B is much smaller than Region A, ensuring that the data from Phase 2 do not overwrite the data from Phase 1. Furthermore, using pseudo-random embedding paths guarantees deterministic reproducibility for authorized parties who hold $K_{S}$, thereby providing an additional security layer.

## 4. Theoretical Analysis

In this section, we present a theoretical analysis of the proposed algorithm. Our analysis focuses on security, embedding capacity, and efficiency. This examination demonstrates how the algorithm improves security and practical performance in distributed steganography.

### 4.1. Security Analysis

The security of the proposed algorithm is significantly enhanced by integrating threshold secret sharing schemes and a dual-layer embedding mechanism. Using secret sharing functions $F_{S1}$ and $F_{S2}$, the algorithm ensures that an adversary must obtain at least *t* message shares and $t^{\prime }$ key shares to reconstruct the secret message *m* and the internal stego key *K*, respectively.

Moreover, the dual-layer embedding creates an additional barrier against steganalytic attacks. Even if an adversary accesses some stego containers, without the shared stego key $K_{S}$, they cannot extract the key shares $k_{i}$ from Phase 2, thereby protecting the message shares $s_{i}$ embedded in Phase 1. The use of non-overlapping embedding regions further ensures data integrity by preventing interference between the two phases, which could otherwise corrupt embedded data and hinder retrieval.

The secrecy of the shared stego key $K_{S}$ is paramount, as it protects the internal stego key *K* and consequently the secret message. Secure key exchange protocols or pre-established secure channels are assumed for the distribution of $K_{S}$ between the sender and the receiver [42,43]. Furthermore, the implementation of stego keys to embed the message and key shares serves to obfuscate the detection and analysis of hidden information by potential attackers, thereby enhancing the algorithm’s resistance to steganalytic techniques.

### 4.2. Embedding Capacity and Efficiency

The embedding capacity and efficiency of the proposed algorithm are significantly influenced by the selection of embedding functions, the choice of secret sharing schemes, and the proportional allocation of embedding regions (proposed in Section 3.4). By matching the embedding regions to the sizes of the message and key shares, the algorithm maximizes the use of the cover media’s capacity while avoiding interference between embedding phases.

In our implementation using image steganography with the Least Significant Bit (LSB) method, both $F_{E1}$ and $F_{E2}$ use LSB embedding with pseudo-random paths determined by *K* and $K_{S}$, respectively. Since each pixel in a grayscale image can hide 1 bit (its LSB), the total embedding capacity *C* of a cover image $C_{i}$ is

(12) C=M×N×c,

where M×N is the dimension of the grayscale cover image, and c=1 bpp (bits per pixel) is the number of bits that can be embedded per pixel.

The sizes of the message share and the key share are denoted by $S_{m}=\left|s_{i}\right|$ and $S_{k}=\left|k_{i}\right|$, respectively, by $S_{k}\ll S_{m}$. Note that $S_{m}$ and $S_{k}$ depend on the chosen secret sharing schemes and can be smaller than the original secret sizes |m| and the internal stego key |K|. For example, certain secret sharing schemes, such as Information Dispersal Algorithms (IDAs), allow the creation of shares that are smaller than the original secret [44]. These schemes trade off some information rate for reduced share sizes, which is advantageous when the embedding capacity is limited.

To ensure that the embedded data fit within the cover image’s capacity, the following condition must be satisfied:

(13) $$S_{m}+S_{k}\leq C.$$

This implies that the cover image must have at least as much capacity as the total size of the data (both message and key shares).

Given the proportional allocation of embedding regions, the capacities for the two regions are

(14) $$C_{A}=\frac{S_{m}}{S_{m}+S_{k}}\cdot C,\;C_{B}=\frac{S_{k}}{S_{m}+S_{k}}\cdot C,$$

where $C_{A}$ and $C_{B}$ are the capacities for Region A (message shares) and Region B (key shares), respectively. This proportional allocation maximizes the utilization of the cover media while maintaining non-overlapping regions.

The embedding efficiency η is defined as the ratio of the total embedded data size to the available capacity:

(15) $$\eta =\frac{S_{m}+S_{k}}{C}.$$

Since c=1 bpp, *C* equals the total number of pixels. The efficiency depends on the amount of capacity used by $S_{m}$ and $S_{k}$. Given that $S_{k}\ll S_{m}$, the overall efficiency can be high. However, to maintain imperceptibility and minimize detection risk, it is advisable to keep η below a certain threshold (e.g., η≤50%).

The algorithm’s efficiency is further improved by

- Selecting secret sharing schemes that generate smaller share sizes (e.g., ramp schemes or IDAs) to reduce the required embedding capacity per container.
- Using a single internal stego key *K* across all containers in Phase 1, which reduces computational overhead and simplifies key management.

In terms of computational complexity, secret sharing schemes $F_{S1}$ and $F_{S2}$, such as Shamir’s Secret Sharing, exhibit the complexities of O(n·t) for secret sharing and $O(t^{2})$ for secret reconstruction, where *t* is the threshold and *n* is the total number of shares. The embedding and extraction functions $F_{E1}$ and $F_{E2}$ have computational complexities proportional to the sizes of $s_{i}$ and $k_{i}$, respectively, denoted as $O(S_{m})$ and $O(S_{k})$. Since $S_{k}\ll S_{m}$ and both $S_{m}$ and $S_{k}$ can be designed to be smaller than |m| and |K|, the computational effort is minimized, enhancing efficiency.

## 5. Evaluation and Results

In this section, an evaluation of the performance and robustness of the proposed distributed steganography algorithm is performed. The assessment includes an analysis of the resilience of the algorithm against known steganographic attacks, imperceptibility, and reliability.

### 5.1. Experimental Setup

In our design, we focus on image steganography for three key reasons: (1) digital images remain the most prevalent cover media in practical steganography due to their high capacity-to-detection-risk ratio; (2) established imperceptibility metrics PSNR (Peak Signal-to-Noise Ratio for distortion measurement) and SSIM (Structural Similarity Index for perceptual quality measurement) allow direct comparison with existing methods; (3) initial validation on images enables benchmarking against state-of-the-art detection tools like StegExpose. Extension to audio/video media, while theoretically feasible, requires specialized adaptations beyond our current scope and will be addressed in future work.

To evaluate the algorithm proposed in Section 3.4, we conduct a series of experiments using the BOSSBase 1.01 image dataset, the standard benchmark in digital image steganography research. This dataset contains 10,000 uncompressed grayscale images of 512×512 pixel resolution that are meticulously crafted for the purpose of benchmark analysis. It offers three key advantages: (1) it provides statistically representative natural image variations useful in real-world applications, (2) it facilitates a standardized comparison with previous work, and (3) it has a large sample size for reliable detection statistics. In our experiments, we use a subset of 3000 images of the 10,000 available in the data set to perform extensive tests under various parameters settings.

Although Section 3.1 shows that the proposed framework can employ multiple steganographic methods in its two phases, the experiments in this study specifically focus on the non-overlapping LSB-based technique for both embedding phases proposed in Section 3.4.

For each experiment, we randomly select *n* images from this pool of 3000. Here, *n* represents both (1) the number of shares in which the secret is split (using Shamir’s Secret Sharing) and (2) the number of cover images in which these shares are embedded. Both the internal stego key *K* and the shared stego key $K_{S}$ are set to 256 bits (32 bytes) long, ensuring a robust foundation for secure embedding. The regions within each cover image designated for embedding are determined using the SHA-3 hash function, seeded with the shared stego key. Secret messages consist of natural language text rather than random bits, simulating real-world usage scenarios where payloads exhibit non-uniform entropy.

The secret message and the internal stego key are divided into *n* shares using Shamir’s Secret Sharing scheme [18], using the same threshold *t* and the number of shares *n* in both embedding phases. In total, 500 experiments are carried out to evaluate the algorithm under different parameter configurations (varying *t*, *n*, secret message length and content). Each experiment consists of both security analysis (measurement of detection rates) and imperceptibility analysis (calculating PSNR and SSIM). In different experiments, we systematically vary the values of *t* and *n*, as well as the length and content of secret messages, to assess their impact on the security and robustness of the algorithm.

To evaluate robustness against known steganographic attacks, we employ StegExpose and Aletheia, which incorporate LSB detectors such as Sample Pair Analysis, RS Attack, Weighted Stego Attack, and Triples Attack [24,25]. These tools measure security through the likelihood of statistical detection. The quality of the stego image after embedding is measured using PSNR and SSIM, providing quantitative metrics to assess the imperceptibility of the embedded data [45].

### 5.2. Security and Steganalysis Detection

We evaluate the robustness of the algorithm against steganalytic attacks using StegoExpose and Aletheia [24,25]. These steganalysis tools employ advanced detection techniques, including the aforementioned methods, which are specifically designed to identify hidden data embedded using LSB approaches. As our algorithm builds on the LSB embedding, we specifically evaluate its resistance against three attacks targeting LSB patterns: (1) *Primary Sets*, which identify deviations in pixel intensity groupings; (2) *Sample Pairs Analysis*, which detects nonrandom LSB transitions through adjacent pixel correlations; and (3) *RS Attack*, which uncovers asymmetries in pixel group flipping. These are selected because they exploit fundamental weaknesses in traditional LSB methods.

Table 1 presents the average results of the StegoExpose analysis for randomly selected images under varying parameters *t*, *n* and message lengths corresponding to the capacity of the cover medium, compared to the results of the steganalysis performed for a simple implementation of the LSB method.

The values of *Primary Sets*, *Sample Pairs*, and *RS Analysis* denote the detection statistics calculated by StegoExpose, with lower values indicating less detectable steganographic content. The *Analyze result* column represents the combined detection score calculated by StegExpose, using a weighted sum of detection statistics. A detection threshold value of 0.15 is used, above which an image is classified as containing steganographic content. The results indicate that none of the stego images produced by the proposed algorithm exceed the stego detection threshold, effectively avoiding detection by StegoExpose under all the conditions tested. In contrast, the traditional LSB method exhibits significantly higher detection scores across all embedding capacities, with multiple instances exceeding the 0.15 threshold in individual tests (despite the average values remaining below it), particularly at 0.9540 bpp where *RS Analysis* reached 0.1143. This demonstrates the superior ability of the proposed method to evade detection compared to conventional LSB embedding.

Furthermore, additional tests performed using Aletheia confirm the algorithm’s robustness, as Aletheia does not detect any stego changes in the generated images. Notably, Aletheia successfully identifies 32% of LSB-embedded stego images in control tests, further emphasizing the security advantages of our two-phase approach. These findings collectively validate the high level of security and undetectability achieved by the proposed method.

### 5.3. Imperceptibility Analysis

To assess the imperceptibility of the embedded data, image quality metrics such as PSNR and SSIM are calculated for each stego image compared to its original cover image. PSNR quantifies the reconstruction quality of the stego image compared to the original cover image, with higher values indicating greater similarity and lower distortion. SSIM assesses the structural similarity between images, focusing on luminance, contrast, and structural information, where values closer to 1 denote a higher similarity.

Table 2 presents the average values of PSNR and SSIM obtained from experiments conducted under various usages of embedding capacity (bpp), threshold values *t*, and number of shares *n*.

Although the classic LSB method shows marginally higher PSNR and SSIM values, this difference is due to the additional security layer of our method. The slight quality reduction (2–3 dB in PSNR and 0.001–0.004 in SSIM) is a deliberate trade-off for enhanced security, as our two-phase approach requires embedding not just payload data but also the secret sharing parameters needed for reconstruction.

**Figure 4:**
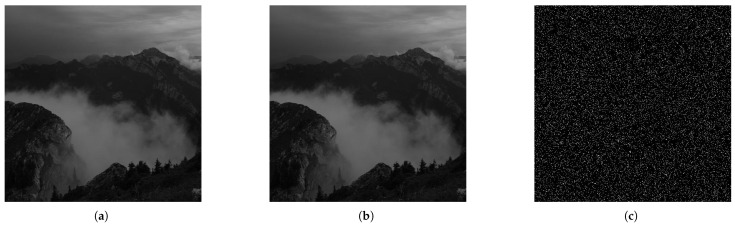
Illustration of the quality assessment for a randomly selected stego image in which a fragment of a secret message has been embedded with capacity over 0.9 bpp. (**a**) The original cover image without any embedded information. (**b**) The corresponding stego image containing the embedded secret message and internal stego key shares. (**c**) A visualization of the pixel-wise differences between the cover and stego images.

The results demonstrate that the proposed distributed algorithm maintains PSNR and SSIM values within acceptable imperceptibility ranges comparable to the standard LSB embedding in different experimental configurations. Specifically, the algorithm exhibits PSNR values consistently above 51 dB and SSIM values around 0.99, with a maximum SSIM deviation of just 0.0043 compared to LSB at full 0.9540 bpp capacity, indicating that the differences between the stego and cover images are negligible and visually imperceptible to the human eye. In particular, even with additional key embedding, all SSIM values remain above 0.9949. The quality and embedding characteristics of the proposed steganographic method are illustrated in Figure 4.

### 5.4. Reliability of Secret Extraction

A critical requirement of any steganographic method is the reliable reconstruction of the hidden secret. In our experiments, with more than 500 independent trials conducted with varying values of *t*, *n*, message lengths, and content, the proposed approach facilitates the lossless extraction of embedded data in each trial. For each experiment, the recovered secret message is compared bit-by-bit with the original embedded payload, confirming a 100% successful recovery rate.

This high recovery performance is inherently related to the deterministic design of the algorithm. Specifically, the use of non-overlapping embedding regions and key-driven pseudo-random embedding paths ensures that no overwrites or collisions occur during the embedding process.

Furthermore, Shamir’s Secret Sharing guarantees that once the threshold *t* is obtained from the *n* shares, the secret can be reconstructed exactly. As a result, authorized users that possess the necessary shares and keys do not encounter information loss, confirming the robustness and determinism of the proposed steganographic framework.

## 6. Discussion

The experimental results presented in the previous section demonstrate the effectiveness and robustness of the proposed distributed steganography algorithm. By integrating a two-phase embedding mechanism with non-overlapping embedding regions and utilizing secret sharing schemes, the algorithm achieves a high level of security and imperceptibility.

The ability of the algorithm to evade detection by advanced steganalysis tools such as StegExpose and Aletheia underscores its robustness against common detection methods targeting LSB embedding techniques. This resistance stems from specific design choices that counter the fundamental assumptions of three primary attacks:

1. *Primary Sets Attack* examines variations in pixel intensity groups to detect hidden payloads. Our algorithm resists this by splitting the secret into multiple shares and distributing each share across pseudo-randomly selected pixels within non-overlapping regions (determined via cryptographic hashing and key-based seeding). This distribution thwarts large, localized modifications and ensures that no single container or region amasses telltale statistical traces of hidden data.
2. *Sample Pairs Analysis* SPA detects embedding by pointing out statistical irregularities in adjacent pixel pairs. We disrupt these patterns through the use of key-driven pseudo-random embedding paths (defined by *K* and $K_{S}$), effectively scattering modified pixels across the image. By preventing clumps of altered pixels, we maintain near-natural adjacency statistics that impede detection by SPA.
3. *RS Attack* identifies hidden data by revealing asymmetries in the pixel group rotation. Our two-phase embedding process strikes a balance between the 0→1 and 1→0 transitions, preserving the symmetry in the modified pixels. When combined with threshold-based secret sharing (so that no single image carries a full payload) and the small portion of cover pixels allocated to embedding the key share, RS detection techniques lose the amplification effect typically exploited to detect skew in flipping patterns.

This multi-layered resistance is particularly significant given that traditional LSB-based steganography is often vulnerable to these statistical attacks due to predictable patterns introduced during data embedding [3]. The use of pseudo-random embedding paths determined by secure keys *K* and $K_{S}$, along with the division of embedding regions, disrupts these patterns, rendering standard detection techniques less effective.

A significant contributing factor to the algorithm’s stealthiness is the combination of embedding strategies employed. While the message and key shares produced by Shamir’s Secret Sharing resemble random bit strings, embedding truly random data into the LSBs can sometimes increase detectability due to disruptions in the natural statistical properties of images. However, our algorithm mitigates this risk by two key mechanisms: (1) the threshold-based secret sharing ensures that no single container contains complete payload information, defeating attacks relying on localized statistical analysis; (2) the distributed embedding mimics natural noise distribution better than concentrated LSB modifications. These design choices ensure that the embedded data are distributed in a manner that preserves the statistical characteristics of the cover images’ LSBs.

By carefully managing the embedding process, the algorithm minimizes detectable anomalies that steganalysis tools, such as StegExpose and Aletheia, typically exploit. The experimental results confirm that, under these conditions, the embedded data remain undetected by advanced steganalysis techniques. This is particularly evident in Table 1, where our method’s *RS analysis* scores remain 35–75% lower than traditional LSB at equivalent capacities, directly reflecting the improved resistance to pixel group analysis attacks. This outcome is directly correlated with previous studies, suggesting that maintaining the statistical properties of the cover medium is crucial to improving the stealth of steganographic methods [46,47].

From a broader perspective, the proposed algorithm makes a significant contribution to the domain of steganography by addressing fundamental limitations of existing techniques. The improved attack resistance demonstrated in Section 5 comes without the typical quality tradeoffs; while our PSNR values are 2–3 dB lower than basic LSB (Table 2), they remain well above the perceptible thresholds. This facilitates streamlined key management through the utilization of a single internal stego key, thus enhancing security without necessitating the use of complex embedding techniques or incurring a substantial computational overhead. The adaptability of the algorithm to different media types and its compatibility with various secret sharing schemes make it a versatile tool for secure communication.

Despite the promising results, several limitations warrant consideration. The reliance on the LSB embedding method, while effective in this context, may still be susceptible to more sophisticated or future steganalysis techniques not covered in this study. However, the foundational resistance mechanisms against Primary Sets, SPA, and RS attacks suggest inherent protection against any method that relies on the localized statistical analysis of LSB patterns. In addition, the size of the secret message is limited by the capacity of the cover media, which may limit the applicability of the algorithm in scenarios requiring the transmission of large amounts of data. Furthermore, the assumption of secure key exchange for the shared stego key $K_{S}$ requires the establishment of a secure channel or prior agreement, which may not always be feasible.

## 7. Conclusions

In this paper, we present a novel distributed steganography algorithm that conceals information between multiple media carriers. Our method integrates secret sharing schemes with a two-phase embedding mechanism that uses non-overlapping regions. This design secures both the secret message and the internal stego key, thereby adding an extra layer of protection against unauthorized access and detection.

Our evaluation shows that the algorithm effectively evades advanced steganalysis tools, such as StegExpose and Aletheia, even when it embeds up to 0.95 bits per pixel. The high PSNR and SSIM values confirm that the modifications introduced remain visually imperceptible, while the secret sharing approach ensures that messages and key shares blend into the cover media like random noise.

Our contributions to the field of steganography include addressing fundamental limitations of existing methods, such as vulnerability to detection, complexity of key management, and lack of adaptability. Using a single internal stego key and a flexible embedding framework, our method simplifies key management and can be adapted to different types of media and secret-sharing schemes.

Future research could focus on exploring alternative secret sharing methods, such as Information Dispersal Algorithms (IDAs), to improve efficiency and reduce share sizes. Furthermore, applying the algorithm to other types of media, including audio and video, and testing it with various embedding techniques will further validate its versatility and robustness. In addition, we plan to incorporate machine learning (ML) and deep learning (DL)-based steganalysis techniques to evaluate and improve the resilience of the algorithm against advanced detection methods. This will provide deeper insight into its security and adaptability in evolving threat landscapes.

In conclusion, our distributed steganography algorithm advances the field by providing a secure, efficient, and adaptable approach to concealing information. Its combination of high imperceptibility and enhanced security against steganalysis makes it a valuable tool for secure communication in a range of applications.

## Figures and Tables

**Table 1:** Average StegoExpose analysis results for the proposed algorithm under different parameters and comparison with the traditional LSB method.

Proposed Method						
Embedding Capacity (bpp)	Threshold *t*	Number of Shares *n*	Primary Sets	Sample Pairs	RS Analysis	Analyze Result
0.4823	2	3	0.0093	0.0083	0.0121	0.0099
	3	5	0.0081	0.0150	0.0184	0.0138
	8	10	0.0157	0.0177	0.0142	0.0199
0.7117	2	3	0.0115	0.0111	0.0137	0.0121
	3	5	0.0583	0.0464	0.0356	0.0467
	8	10	0.0297	0.0226	0.0266	0.0262
0.9540	2	3	0.0333	0.0117	0.0145	0.0199
	3	5	0.0467	0.0533	0.0337	0.0463
	8	10	0.0422	0.0347	0.0294	0.0354
**LSB method**						
0.4823			0.0149	0.0207	0.0194	0.0168
0.7117			0.0295	0.0340	0.0311	0.0415
0.9540			0.0927	0.1035	0.1143	0.0790

**Table 2:** Average image quality metrics for the proposed algorithm under different parameters and comparison with the traditional LSB method.

Proposed Method				
Embedding Capacity (bpp)	Threshold *t*	Number of Shares *n*	PSNR (dB)	SSIM
0.4823	2	3	54.3757	0.9984
	3	5	54.4042	0.9982
	8	10	54.3718	0.9983
0.7117	2	3	52.6166	0.9981
	3	5	52.1143	0.9974
	8	10	52.2383	0.9969
0.9540	2	3	51.4060	0.9949
	3	5	51.4015	0.9963
	8	10	51.4158	0.9968
**LSB method**				
0.4823			56.1315	0.9997
0.7117			54.0824	0.9994
0.9540			53.1121	0.9992

## Data Availability

The original contributions presented in this study are included in the article. Further inquiries can be directed to the corresponding author.

## Abbreviations

The following abbreviations are used in this manuscript:

LSB	Least Significant Bit
PSNR	Peak Signal-to-Noise Ratio
SSIM	Structural Similarity Index Measure
DCT	Discrete Cosine Transform
DWT	Discrete Wavelet Transform
IDAs	Information Dispersal Algorithms
